# Determinant Factors of Untreated Dental Caries and Lesion Activity in Preschool Children Using ICDAS

**DOI:** 10.1371/journal.pone.0150116

**Published:** 2016-02-22

**Authors:** Tássia Cristina de Almeida Pinto-Sarmento, Mauro Henrique Abreu, Monalisa Cesarino Gomes, Edja Maria Melo de Brito Costa, Carolina Castro Martins, Ana Flávia Granville-Garcia, Saul Martins Paiva

**Affiliations:** 1 Department of Dentistry, State University of Paraíba, Campina Grande, Paraíba, Brazil; 2 Department of Public Health, Federal University of Minas Gerais, Belo Horizonte, Minas Gerais, Brazil; 3 Department of Paediatric Dentistry and Orthodontics, Federal University of Minas Gerais, Belo Horizonte, Minas Gerais, Brazil; Navodaya Dental College and Hospital, Mantralayam Road, INDIA

## Abstract

The aim of the present study was to investigate determinant factors associated with the presence of dental caries and lesion activity in preschool children. A population-based, cross-sectional study was carried out with 843 children of aged three to five years enrolled at public and private preschools in the city of Campina Grande, Brazil. A questionnaire addressing socio-demographic data and oral health care was self-administered by parents/caregivers. Three dentists previously calibrated examined the children for the diagnosis of dental caries and lesion activity using the International Caries Detection and Assessment System (ICDAS). Nutritional status was evaluated based on the body mass index. Logistic regression analysis for complex samples was performed (α = 5%). The prevalence of dental caries was 66.3%. Among the children with caries, 88.0% had active lesions. Dental caries was more prevalent in girls (OR = 1.53, 95%CI: 1.05–2.23), in children from families with a monthly household income ≤US$312.50 (OR = 2.38, 95%CI: 1.65–3.43) and those whose mothers had up to eight years of schooling (OR = 1.55, 95%CI: 1.07–2.23). Lesion activity was significantly associated with mother’s schooling ≤ 8 years (OR = 2.15, 95%CI: 1.15–4.00). The prevalence rates of dental caries and lesion activity were high and mainly associated with a lower socioeconomic status and mother’s schooling.

## Introduction

Despite the decline in tooth decay in the paediatric population throughout the world, dental caries remains the most common childhood disease, causing pain, chewing difficulties, general health disorders, psychological problems and a substantial impact on quality of life [1]. Thus, dental caries is an international public health problem and early detection is important to establishing adequate preventive measures as well as avoiding premature treatment and consequent costs [2–4].

Dental caries has traditionally been assessed using the criteria established by the World Health Organization (WHO) in 1997, which include only obvious carious lesions [5]. However, the detection of lesions in the early non-cavitated stage is important to the diagnostic process [2,6]. An international group of researchers designed the International Caries Detection and Assessment System (ICDAS) in 2007 to allow the assessment of carious lesions in the enamel and active lesions in the dentine. This system is based on the combined knowledge of the clinical appearance of the lesion, whether the lesion is in a plaque stagnation area and tactile sensation (texture) when a round-tipped probe is gently drawn across the surface of the tooth [7,8]. The ICDAS has proven to be reliable, offering increased sensitivity and accuracy in detecting carious lesions, and is indicated for epidemiological surveys involving children [5,9,10].

Despite recent improvements in the oral health of children in Brazil, dental caries remains highly prevalent, with persisting inequalities. The mapping of patterns of oral health in young children and a better understanding of caries initiation and progression are essential to understanding the behaviour of caries as well as determining the best way to control and prevent this disease [11,12]. The detection of early dental caries is one of the most accurate measures for predicting children at a risk for tooth decay [13]. Teeth can develop dental caries as early as three to six months following eruption [14]. Lesion activity at 18 months is related to the incidence of caries at the age of two and three years [15]. Risk factors associated with lesion activity at this age may be related to breast-feeding, bottle feeding, sucrose intake and the absence of tooth brushing performed by a caregiver [15]. Other risk factors include dietary habits, the transmission of pathological microorganisms, especially from the caregiver, oral hygiene habits, family values, traditions and lifestyles [16]. Moreover, the factor nutritional status has not been adequately investigated [17]. Thus, there is a need for a better understanding of the risk factors related to the prevalence of dental caries as well as the best manner to address these factors [4,18].

The assessment of lesion activity is essential to the diagnosis and appropriate treatment [19]. The detection of lesion activity allows the establishment of greater or lesser invasive treatment [20]. From the public health standpoint, minimally invasive treatment reduces treatment costs, as inactive caries can be treated with prophylactic rather than restorative measures [21]. Only one study has used the ICDAS to evaluate lesion activity [22]. However, the study cited was conducted on the permanent dentition. Although studies have assessed risk factors of lesion activity in the primary dentition [15], this is the first study to investigate this association using the ICDAS as the diagnostic criteria for dental caries. While it is expected that the same risk factors exert an influence on dental caries and lesion activity, the lack of previous studies employing the ICDAS for the primary dentition justifies the present investigation.

The aim of the present study was to determine factors associated with dental caries and lesion activity in the primary dentition using the ICDAS as the diagnostic criteria.

## Materials and Methods

### Ethical considerations

This study received approval from the Human Ethics Research Committee of the State University of Paraíba (00460133000–11) in compliance with Brazilian National Health Council Resolution 196/96. The study was conducted in accordance with the ethical standards laid down in the 1964 Declaration of Helsinki. All parents/guardians received information regarding the objectives of the study and signed a statement of informed consent.

### Sample characteristics

A school-based, cross-sectional study was carried out involving boys and girls aged three to five years enrolled at private and public preschools in the city of Campina Grande, Brazil. Campina Grande (population: 400,002) is an industrialised city in northeast Brazil and is divided into six health districts, with 22,400 estimated inhabitants aged three to five years [23]. The city has considerable cultural, social and economic disparities, with a mean income of US$110 *per capita* and a Human Development Index of 0.72 [23]. The participants were selected from a total population of 12,705 preschool children in this age group, corresponding to 6.6% of the population and therefore representative of preschool children in Campina Grande.

The sample size was calculated with a 4% margin of error, a 95% confidence level and a 50.0% prevalence rate of dental caries. As no previous study have been conducted to determine the prevalence of dental caries among young children in the city of Campina Grande, a rate of 50% was considered to increase the statistical power [24]. A correction factor of 1.2 was applied to compensate for the design effect. The minimum sample size was estimated at 720 schoolchildren. A further 20% was added to compensate for possible losses, giving a total sample of 864 schoolchildren. A two-phase sampling method was used to ensure representativeness. The percentage distribution of three-to-five-year-old preschool children in each administrative district was calculated from information provided by the Municipal Secretary of Education of Campina Grande. The sample was stratified according to administrative district and type of institution. In the first phase, preschools were randomly selected from each administrative district using the Microsoft Excel program (Microsoft Corp., version 2003, USA). Thirty-three preschools were randomly selected from each of the six health districts in the first phase (18 of the 127 public preschools and 15 of the 122 private preschools). In the second phase, children from each school were randomly selected from a list of names of all the students. Children were numbered based on the list and the numbers were written on pieces of paper, which were then randomly chosen by lots. The ratio of the total population enrolled in private and public preschools in each administrative district of the city was maintained in the sample distribution.

### Eligibility criteria

The following were the inclusion criteria: age ranging from 36 to 71 months; enrolment in preschool; absence of systemic disease (according to parents’/caregivers’ reports); and exclusively in the primary dentition phase.

### Training and calibration exercise

The calibration exercise consisted of two stages. The theoretical stage involved a discussion of the ICDAS diagnostic criteria and lesion activity as well as an analysis of photographs. A specialist in paediatric dentistry (SMP) was the gold standard in the theoretical framework and coordinated this step, instructing three general dentists on how to perform the examination. The second step was the clinical stage performed in a randomly selected public preschool that was not part of the main sample. Each dentist examined 50 previously selected children between 36 and 71 months of age. Inter-examiner agreement was tested by comparing each examiner with the gold standard. Thirty children were re-examined after a seven-day interval for determination of intra-examiner agreement. Data analysis involved the calculation of Cohen’s Kappa coefficient on a tooth-by-tooth basis (K = 0.85 to 0.90 for both inter-examiner and intra-examiner agreement). As the Kappa coefficients were very good [25], the examiners were considered able to perform the epidemiological study.

### Pilot study

A pilot study was conducted to test the methodology and comprehension of the questionnaire. The children in the pilot study (n = 40) were not included in the main sample. The results revealed no misunderstandings regarding the questionnaire.

### Non-clinical data collection

The parents/caregivers were asked fill out a questionnaire with two sections: 1) socio-demographic data (parent’s age, parent’s schooling in number of years of study, number of persons residing in the home, type of school, relationship of respondent to the child and monthly household income); and 2) dental care (tooth brushing frequency and person responsible for the child’s tooth brushing).

Parent’s schooling was dichotomized as ≤ eight years of schooling and > eight years of schooling. This cut-off point was chosen because it was the median in the sample and eight years of study corresponds to primary school in the Brazilian education system. Household income was dichotomized based on the monthly minimum wage in Brazil (= U$312.50), which was the median in the sample.

### Clinical data collection

The clinical examination was performed after the return of the signed statement of informed consent and questionnaires. Oral examinations were performed by three dentists who had undergone the calibration process. The children cleaned their teeth prior to the clinical examination to remove bacterial biofilm from the tooth surfaces using a kit containing a toothbrush, fluoridated toothpaste and dental floss. The examinations were performed by the dentists at the preschools with of the preschool children seated in front of the examiner with aid of a portable lamp attached to the examiner’s head (Tikkina 2, Petzl, Rawang, Malaysia). Individual cross-infection protection equipment was used. Packaged and sterilised disposable mouth mirrors (PRISMA®, São Paulo, SP, Brazil), WHO probes (Golgran Ind. e Com. Ltda., São Paulo, SP, Brazil) and dental gauze (to dry the teeth) were used for the examination.

The ICDAS criteria were employed [7]: 0) sound; 1) first visual change in enamel (due to the epidemiological nature of the present study, code 1 was not used, as drying of the teeth was performed with gauze rather than compressed air); 2) distinct visual change in enamel when wet; 3) localised enamel breakdown (without clinical visual signs of dentine involvement); 4) underlying dark shadow from dentine; 5) distinct cavity with visible dentine; and 6) extensive distinct cavity with visible dentine. A score of 0 was considered normal. A score of 2 was considered caries restricted to the enamel without dentine involvement. Cavitated teeth were considered those with scores ≥ 3, as rupture of the structure of the enamel surface is seen beginning at this point. Scores of 5 and 6 were considered severe dental caries, as these scores determine obvious cavitation extending through the dentine. Dental caries was recorded when at least one tooth received code 2 or higher.

Lesion activity was considered as follows: 1) enamel–the lesion is whitish/yellowish; the lesion is chalky (lack of lustre); the lesion may be cavitated or not; the lesion feels rough upon probing; probing may or may not lead to the discovery of a cavity; 2) dentine–the lesion may manifest itself as a shadow below the intact but demineralized enamel; if a cavity extends into the dentine, the dentine appears yellowish/brownish; dentine soft upon probing [26]. Lesion activity was recorded when at least one active carious lesion was found.

For dental caries and lesion activity, each individual was considered a unit of analysis. As the region in which the study was conducted does not have a policy of a fluoridated water supply, a fluoride varnish (Duraphat®—5% NaF) was applied to all children after the examinations and those with carious lesions were sent for dental treatment.

### Anthropometric data

Nutritional status was determined using the Anthroplus software programme, which provides the body mass index (BMI) following the input of weight (kilograms), height (meters), date of birth and date of the clinical examination [27]. Z scores were used to determine nutritional status based on standard deviations (SD) of the BMI. Children with a Z score < -1 for their height/weight ratio were classified with malnutrition/severe malnutrition; children with a score of -1 SD ≥ Z ≤ +1 SD for their height/weight ratio were considered in the ideal range; children with a score of + 1 SD ≥ Z ≤ 3 SD for their height/weight were considered overweight/obese.

### Statistical analysis

The frequency distribution of the data was determined. Dental caries and lesion activity were the dependent variables. Logistic regression considering the design effect in complex samples (considering sample weights) was conducted for each dependent variable (p < 0.05). Effect sizes were designated as small (< 0.20), moderate (0.20 to 0.70) or large (> 0.70) [28]. Independent variables with a p-value < 0.20 were incorporated into the multiple logistic regression stepwise model. Statistical analysis was carried out using the Statistical Package for Social Sciences (SPSS for Windows, version 18.0, SPSS Inc, Chicago, IL, USA).

## Results

Eight hundred forty-three pairs of parents/guardians and children took part in the present study. The response rate was 97.56%. The loss of 21 pairs was due to the non-participation of the child for medical reasons (n = 1), incomplete questionnaires (n = 10), absence from preschool on the days scheduled for the clinical examination (n = 5) and a lack of cooperation during the examinations (n = 5).

Boys accounted for 51.7% of the sample and girls accounted for 48.3%. The children were balanced by age: 32.6% were three years old, 39.6% were four years old and 27.8% were five years old. The majority of mothers (53.8%) had more than eight years of study. Fifty-five percent of families had a monthly household income greater than or equal to US$ 312.50. The vast majority (84.4%) of families had less than six residents in the home. A total of 31.3% of the participants were the only child in the family, 41.6% were the youngest child, 14.7% were the oldest child and 12.4% were the middle child. A total of 45.9% were enrolled at private preschools and 54.1% were enrolled at public preschools (Table 1).

**Table 1:** Sample characterisation.

Variables	Frequency	
	n	(%)
**Sex of child**		
Boys	407	48.3
Girls	436	51.7
**Age of child**		
3	275	32.6
4	334	39.6
5	234	27.8
**Mother’s schooling (years)** *		
> 8 years	452	53.8
≤ 8 years	388	46.2
Monthly household income*		
≥ US$ 312.50	442	55.0
< US$ 312.50	362	45.0
Number of residents in home*		
< 6	699	84.4
≥ 6	129	15.6
Child’s birth order*		
Only child	263	31.3
Younger child	349	41.6
Older child	123	14.7
Middle child	104	12.4
**Type of preschool**		
Private	387	45.9
Public	456	54.1
**Dental Caries**		
Yes	559	66.3
No	284	33.7
**Lesion activity**		
Active	492	88.0
Inactive	67	12.0

*Sample less than 843 due to the failure of some interviewees to provide this information.

The prevalence of dental caries was 66.3%. Among the children with caries, 88.0% had active lesions (Table 1). Among the teeth with dental caries, 24.0% were non-cavitated (score of 2), 76.0% were cavitated (scores 3 to 6) and 63.0% were severely cavitated (scores 5 and 6).

Table 2 displays the logistic regression for predictors of dental caries. In the final model, the girls (OR = 1.53, 95% CI: 1.05 to 2.23; effect size: 0.06), mother’s schooling ≤ 8 years (OR = 1.55, 95% CI: 1.07 to 2.23; effect size: 0.15) and monthly household income ≤ U$ 312.50 (OR = 2.38, 95% CI: 1.65 to 3.43; effect size: 0.20) were significant predictors of dental caries.

**Table 2:** Logistic regression model considering design effect (unadjusted and adjusted odds ratio) for dental caries and independent variables among children aged 3 to 5 years (OR, 95% CI).

Variables	Dental caries		Unadjusted bivariate	Adjusted multivariate		Effect size*
	Yes	No	Model	Model**		
	n (%)	n (%)	OR (95%CI)	p-value	OR (95%CI)	
**Sex of child**						
Boys	277(63.5)	159(36.5)	1.00		1.00	
Girls	282(69.3)	125(30.7)	1.39 (0.97–1.99)	0.028	1.53 (1.05–2.23)	0.06
**Age of child**						
3 years	168(61.1)	107(38.9)	1.00	**-**	**-**	-
4 years	231(69.2)	103(30.8)	0.79 (0.53–1.17)	**-**	**-**	
5 years	160(68.4)	74(31.6)	1.09 (0.68–1.74)	**-**	**-**	-
**Mother’s schooling (years)**						
> 8 years	268(59.3)	184(40.7)	1.00		1.00	
≤ 8 years	288(74.2)	100(25.8)	2.29 (1.62–3.32)	0.019	1.55 (1.07–2.23)	0.15
**Monthly household income**						
> U$ 312.50	204(56.4)	158(43.6)	1.00		1.00	
≤ U$ 312.50	334(75.6)	108(24.4)	2.90 (2.03–4.16)	<0.001	2.38 (1.65–3.43)	0.20
**Number of residents in home**						
< 6	448(64.1)	251(35.9)	1.00	-	-	-
≥ 6	101(78.3)	28(21.7)	1.79 (1.09–3.11)	-	-	
**Child’s birth order**						
Only child	101(38.4)	162(61.6)	1.00	-	-	
Youngest child	234(67.0)	115(33.0)	1.24 (0.82–1.89)	-	-	-
Oldest child	84(68.3)	39(31.7)	1.41 (0.80–2.47)	-	-	
Middle child	75(72.1)	29(27.9)	1.32 (0.70–2.49)	-	-	
**Tooth brushing frequency**						
≥ 2 times/day	482(65.8)	250(34.2)	1.00	-	-	
< 2 times/day	72(70.6)	30(29.4)	1.22 (0.70–2.12)	-	-	-
**Who brushes child’s teeth**						
Mother	386 (63.8)	219 (36.2)	1.0	-	-	
Other adult	15(60.0)	10(40.0)	1.26 (0.49–3.22)	-	-	-
Child	151(73.3)	55(26.7)	1.44 (0.92–2.24)	-	-	
**Nutritional status**						
Overweight/ obese	151(60.9)	97(39.1)	1.00	-	-	
Ideal range	366(67.9)	173(32.1)	1.50 (1.01–2.22)	-	-	-
Malnutrition/severe malnutrition	42(75.0)	14(25.0)	1.46 (0.63–3.36)	-	-	

*Chi-square test with α set at 5.0%.

**Variables incorporated into multivariate model (p<0.20): sex of child, mother’s schooling, monthly household income, number of residents in home, who brushes child’s teeth, nutritional status.

Table 3 displays the logistic regression for predictors of lesion activity. In the final model, only mother’s schooling ≤ 8 years (OR = 2.15, 95% CI: 1.15 to 4.00; effect size: 0.10) was significant predictor of lesion activity.

**Table 3:** Logistic regression model considering design effect (unadjusted and adjusted odds ratio) for lesion activity and independent variables among children aged 3 to 5 years (OR, 95% CI).

Variables	Lesion activity		Unadjusted bivariate	Adjusted multivariate		Effect Size*
	Active	Active	model	model**		
	n (%)	n (%)	OR (95%CI)	p-value	OR (95%CI)	
**Gender of child**						
Boys	245(88.4)	32(11.6)	1.00	-	-	
Girls	247(87.6)	35(12.4)	1.12 (0.59–2.13)	-	-	-
**Age of child**						
3 years	144(85.7)	24(14.3)	0.73 (0.31–1.72)	**-**	**-**	-
4 years	208(90.0)	23(10.0)	1.41 (0.62–3.18)	**-**	**-**	
5 years	140(87.5)	20(12.5)	1.00	**-**	**-**	
**Mother’s schooling (years)**						
> 8 years	227(84.7)	41(15.3)	1.00		1.00	0.10
≤ 8 years	263(91.3)	25(8.7)	2.27 (1.23–4.17)	0.017	2.15 (1.15–4.00)	
**Monthly household income**						
> U$ 312.50	176(86.3)	28(13.7)	1.00	-	-	
≤ U$ 312.50	299(89.5)	35(10.5)	1.83 (0.96–3.50)	-	-	-
**Number of residents in home**						
< 6 people	393(87.7)	55(12.3)	1.00	-	-	-
≥ 6 people	89(88.1)	12(11.9)	1.23 (0.60–2.53)	-	-	
**Child’s birth order**						
Only child	137(84.6)	25(15.4)	1.00	-	-	
Youngest	212(90.6)	22(9.4)	2.12 (0.97–4.64)	-	-	-
Oldest	72(85.7)	12(14.3)	2.00 (0.86–4.67)	-	-	
Middle child	67(89.3)	8(10.7)	1.94 (0.78–4.86)	-	-	
**Tooth brushing frequency**						
≥ 2 times/day	421(87.3)	61(12.7)	1.00	-	-	
< 2 times/day	66(91.7)	6(8.3)	1.36 (0.53–3.53)	-	-	-
**Who brushes child’s teeth**						
Mother	336 (87.0)	50(13.0)	1.00	-	-	
Other adult	14(93.3)	1(6.7)	0.44 (0.06–3.53)	-	-	-
Child	135(89.4)	16(10.6)	1.60 (0.82–3.11)	-	-	
**Nutritional status**						
Overweight/ obese	130(86.1)	21(13.9)	1.00	-	-	
Ideal range	326(89.1)	40(10.9)	1.53 (0.75–3.10)	-	-	-
Malnutrition/severe malnutrition	36(85.7)	6(14.3)	1.37 (0.47–4.02)	-	-	

*Chi-square test with α set at 5.0%.

**Variables incorporated into multivariate model (p<0.20): mother’s schooling, monthly household income, child’s birth order, who brushes the child’s teeth.

Methods: logistic regression considering design effect in complex sample.

## Discussion

The present school-based study sought to identify possible determinant factors associated with the presence of dental caries and disease activity in preschoolers using the ICDAS as the diagnostic method. Studies have used the WHO criteria for the diagnosis of dental caries diagnosis [29–31], which do not discriminate initial carious lesions or lesion activity. In the present study, mother’s schooling was the only risk factor for both dental caries and caries activity in preschool children.

The population studied had a high prevalence rate of dental caries (66.3%), which means that only one third did not need treatment beyond preventive measures. Moreover, 63% of the children with caries had severe degrees of the disease, such as a distinct, extensive cavity with visible dentine [7]. Such results indicate that these children needed some kind of operative dentistry. However, the mere diagnosis of dental caries does not indicate caries activity. Thus, the determination of lesion activity allows differentiating children in need of dental treatment from those not in need of treatment. Among the entire sample, 58.4% (492 children) needed some type of treatment (active dental caries in enamel or dentine). Not all active lesions in enamel will progress to more severe degrees and many may go into remission. Such lesions can be treated conservatively with fluoride therapy. On the other hand, if not treated, active lesions in enamel may progress to the dentine and require restoration. Moreover, active lesions in the dentine cannot go untreated due to the risk of severe complications, which occur in approximately 40% of cases [14]. Active cavitated lesions in the dentine can involve the pulp and require endodontic treatment or extraction, which are more complex procedures. Moreover, controlling caries at this point implies efforts beyond operative dentistry, such as sugar control, fluoride treatment, a change in habits and family involvement.

Girls had a greater likelihood of having dental caries, which is in agreement with findings described in previous studies [32,33]. This may be explained by the earlier eruption of teeth in girls, with a consequent longer exposure to cariogenic factors in the oral environment [34]. Otherwise, sex was not associated with lesion activity. The exposure to fluoride may play a role in the remineralisation of dental caries equally in both sexes.

Dental caries and lesion activity had one common risk factor in the present investigation: mother’s schooling. Theses associations may be explained by a greater offer of sweets and diets with sugar by mothers with a lower level of schooling [35] and a lack of knowledge regarding the influence of diet on the progression of dental caries [12]. Moreover, household income was a significant predictor of caries, which is in agreement with findings reported in the literature [16,35,36]. This underscores social inequalities with regard to dental caries, the prevalence of which is higher among individuals with a low income and low level of schooling, confirming that individuals with a low socioeconomic status are more exposed to a various risk factors that affect oral health [37,38]. The association between lesion activity and income should be studied in other populations.

Differences among social classes constitute a huge problem in Brazil, where a large portion of the population has a low income and the minority is rich. Median monthly household income was equivalent to U$312.50 (the minimum wage in Brazil). Only 50% of the sample earned more than the minimum wage and income distribution was very irregular. This shows that a large portion of the children lived in families with a low income and their mothers had a lower education level, which may explain the high prevalence rates of dental caries and lesion activity and also underscores social inequalities with regard to these outcomes.

One problem regards the percentage of children not enrolled at preschools. Among the 22,440 children aged three to five years in the city of Campina Grande, 12,705 were enrolled at preschools. This problem is common throughout Brazil. If the remaining children were considered in the prevalence study of dental caries, the frequency would have been much greater than that presented herein.

This study has limitations that should be addressed, such as information bias regarding income and memory bias, which is a common occurrence when data are collected retrospectively. Another limitation regards the lack of the detection of the first visual change in enamel (ICDAS code 1) [36], which may have underestimated the prevalence of dental caries. Moreover, no dietary histories of the patients through diet recalls were taken.

The prevalence rates of dental caries and lesion activity were high in the present sample. A low level of mother’s schooling was a common risk factor for both outcomes, whereas a lower income and the girls were risk factors for dental caries. In Brazil, there is a lack of specialized care for infants and preschoolers at public health services [39]. Thus, current oral health policies are inadequate. The present findings highlight the need for policy revision to include early childhood in preventive dental strategies and define appropriate prevention approaches. Other authors report that counselling mothers in the initial days following the birth of a child regarding oral health care, with the presentation of educational material, is an effective approach to improving oral health in preschool children [31].

## Supporting Information

S1 File Article “Perceived impact of dental pain on the quality of life of preschool children and their families” (PONE-D-14-46591). (DOC)
