# Supplementary material for: Determinant Factors of Untreated Dental Caries and Lesion Activity in Preschool Children Using ICDAS
Source: PLoS One. 2016 Feb 22;11(2):e0150116. doi: 10.1371/journal.pone.0150116 (PMC4763475; doi:10.1371/journal.pone.0150116)
Supplement: S1 File — (DOC) [file pone.0150116.s001.doc]

**Perceived impact of dental pain on the quality of life of preschool children and their families**

Marayza Alves [Clementino]^1^, Monalisa Cesarino [Gomes]^1^, Tássia Cristina de Almeida [Pinto-Sarmento]^1^, Carolina Castro [Martins]^2^, Ana Flávia [Granville-Garcia]^1*^, Saul Martins [Paiva]^2^

^1^ Department of Dentistry, State University of Paraíba, Campina Grande, Brazil

^2^ Department of Pediatric Dentistry and Orthodontics, Dental School, Federal University of Minas Gerais, Belo Horizonte, Brazil

* Corresponding author

E-mail: anaflaviagg@hotmail.com

**Abstract**

The aim of the present study was to evaluate the perceived impact of dental caries and dental pain on oral health-related quality of life (OHRQoL) among preschool children and their families. A cross-sectional study was conduct with 843 preschool children in Campina Grande, Brazil. Parents/caregivers answered a questionnaire on socio-demographic information, their child’s general/oral health and history of dental pain. The Brazilian version of the Early Childhood Oral Health Impact Scale was administered to determine the perceived impact of caries and dental pain on OHRQoL. The children underwent an oral examination. Logistic regression for complex sample was used to determine associations between the dependent and independent variables (OR: Odds ratio, α = 5%). The independents variables that had a p-value <0.20 in the bivariate analysis were selected for the multivariate model. The prevalence of dental caries and dental pain was 66.3% and 9.4%, respectively. Order of birth of the child, being the middle child (OR: 10.107, 95%CI: 2.008-50.869) and youngest child (OR: 3.276, 95%CI: 1.048-10.284) and dental pain (OR: 84.477, 95%CI: 33.076-215.759) were significant predictors of the perceived impact on OHRQOL for children. Poor perception of oral health was significant predictor of the perceived impact on OHRQOL for family (OR=7.397, 95%CI: 2.190-24.987). Dental caries was not associated with a perceived impact on the ORHQoL of either the children or their families. However, order of child birth and dental pain were indicators of impact of OHRQoL on preschool children and poor perception of oral health was indicators of impact on families.

**Introduction**

Dental caries (tooth decay) is one of the most prevalent chronic childhood diseases worldwide and is a major problem both from the public health perspective and for individual families who have to deal with a young child suffering from dental pain [1]. This condition often goes untreated in young children [2].

Carious lesions are among the major oral health problems of preschool child, even though if in this age group, children lose the first set of teeth. Oral health problems can cause difficulties in chewing, decreased appetite, loss of weight, sleep disturbances, behavioral changes and poorer school performance, which leads to a poorer quality of life [3]. In addition, traumatic dental injury and presence malocclusion in this age group has also been the focus of studies assessing the quality of life, as the cases of dental trauma, often neglected by their parents/caregivers. Preschool children may also suffer from dental pain and eruption disturbances. Measuring OHRQoL can make an important contribution by providing more data on this issue to help guide the oral health policies [4-5].

The consequences of untreated caries affect the oral health-related quality of life (OHRQoL) of children and their families due to dental pain and esthetic issues [4]. Moreover, caries can lead to psychosocial problems, impaired speech, the development of parafunctional habits, the loss of vertical dimension and impaired chewing capacity [4-6].

Studies stress the importance of considering the functional and psychosocial dimensions of oral health for the implementation and evaluation of dental interventions within the public health realm [1,2,4]. A number of assessment tools have been developed to measure the impact of oral problems on quality of life. The Early Childhood Oral Health Impact Scale (ECOHIS) was created for the assessment of ORHQoL among children aged three to five years. This questionnaire is answered by parents / caregivers and is not based on self-report of preschoolers. At this age, children have difficulty understanding basic concepts of health, are unable to express themselves adequately to provide some answers[3,7,8]. The Brazilian version of this questionnaire (B-ECOHIS) has been validated in Portuguese for use in Brazil and has been employed in previous studies [9,10].

To date, no studies have addressed the perceived impact of dental caries and dental pain on OHRQoL of children aged three to five years using the International Dental Caries Detection and Assessment System (ICDAS-II) and the B-ECOHIS. A single study evaluated OHRQoL using the B-ECOHIS and dental caries using the ICDAS-II on a sample of children aged six and seven years [4]. However, the ECOHIS was designed for use on the three-to-five-year-old age group [7,9].

The aim of the present study was to evaluate the perceived impact of dental caries and dental pain on oral health-related quality of life (OHRQoL) among preschool children and their families.

**Materials and methods**

**Sample characteristics**

The present study received approval from the Human Research Ethics Committee of the State University of Paraiba (Brazil) under process number 00460133000-11 in compliance with Resolution 196/96 of the Brazilian National Health Council.

A school-based, cross-sectional study was carried out involving male and female children aged three to five years enrolled at private and public preschools in the city of Campina Grande, Brazil. Campina Grande is an industrialized city in northeastern Brazil with a population of 386,000 inhabitants and is divided into six administrative districts. The city has considerable cultural, social and economic disparities, with a mean monthly income per capita equal to US$ 110 and a Human Development Index of 0.72 [11]. The participants were selected from a total population of 12,705 children in this age group and corresponded to 6.41% of the entire populationand therefore representative of preschool children in Campina Grande.

A two-phase sampling method was used to ensure representativeness. A list of preschools was obtained from the Municipal Secretary of Education of Campina Grande.To ensure the representativeness a sample calculation was done. Through a stratified sampling procedure, preschools were selected at draw from each district of Campina Grande. Then the number of schoolchildren from each preschool was proportional to the number of schoolchildren enrolled in each district. The sample was obtained from the proportion estimation calculation. Preschools were randomly selected by draw from each district in the first phase and children were randomly selected from each preschool in the second phase. Eighteen of the 127 public preschools and 15 of the 122 private preschools were randomly selected by lots. The sample size was calculated with a 4% margin of error, a 95% confidence level and a 50% prevalence rate of perceived impact on child and family OHRQoL. A correction factor of 1.2 was applied to compensate for the design effect [12].

The minimum sample size was estimated at 720 schoolchildren, to which an additional 20% was added to compensate for possible losses, giving a total sample of 864 schoolchildren. The registration of the study with Clinicaltrial.gov is NCT02443207.

**Eligibility criteria**

The inclusion criteria were age three to five years of age, enrollment in preschool or daycare and free of any systemic disease according to parents’/caregivers’ reports. Parental authorization was required and was obtained through a signed statement of informed consent.

**Training and calibration exercise**

The calibration exercise consisted of two steps (theoretical and clinical). The theoretical step involved a discussion of the criteria for the diagnosis of dental caries, TDI, malocclusion and an analysis of photographs. A specialist in pediatric dentistry (gold standard in this theoretical framework) coordinated this step, instructing three general dentists on how to perform the examination. The clinical step was performed at a randomly selected preschool that was not part of the main sample. Each dentist examined 50 previously selected children between three and five years of age. Inter-examiner agreement was tested by comparing each examiner with the gold standard (K = 0.85 to 0.90). A seven-day interval was respected between clinical examinations for the determination of intra-examiner agreement (K = 0.85 to 0.90). Data analysis involved Cohen’s Kappa coefficient on a tooth-by-tooth basis. As Kappa coefficients were very good [13], the examiners were considered capable of performing the epidemiological study.

**Study pilot**

A pilot study was conducted to test the methodology and comprehension of the questionnaires. The children in the pilot study (n = 40) were not included in the main sample. As there were no misunderstandings regarding the questionnaires or the methodology, no changes to the data collection process were deemed necessary.

**Non-clinical data collection**

The collection of the non-clinical data involved the B-ECOHIS and questionnaires addressing socio-demographic data, parents’/caregivers’ perceptions of their child’s general and oral health and a history of dental pain. All questionnaires were filled out by the parents/caregivers.

The B-ECOHIS addresses the perceptions of parents/caregivers of the perceived impact of oral health problems on the quality of life of preschool children and their families. This questionnaireis divided into two sections (Child Impact and Family Impact), six domainsand thirteen questions. The domains of the ‘Child Impact’ section are symptoms (1 item), function (4 items), psychology (2 items) and self-image/social interaction (2 items). The domains of the ‘Family Impact’ section are parental distress (2 items) and family function (2 items). Each item has six response options: never; hardly ever; sometimes; often; very often; and “I don’t know” (“don’t know” responses are not considered). In the present study, the perceived impact on the OHRQoL of child and family were the dependent variables. Perceived impact on child and family was recorded when at least one response of “sometimes”, “often” or “very often” was chosen, meaning presence of impact (yes). That means that the independent variables can bring complications and cause presence of perceived impact on OHRQoL. The responses of “never” and “hardly ever” were considered indicative of an absence of impact (no) [7,9].

A questionnaire addressing the following socio-demographic variables was administered: sex and age of child; parent’s/caregiver’s age and schooling; type of preschool (public or private); number of residents in the home; child’s birth order among siblings; household income (classified based on the Brazilian monthly minimum wage = US$312.50), parents’/caregivers’ perception of their child’s general and oral health; and a history of dental pain.

**Clinical data collection**

After the return of the questionnaires and signed statement of informed consent, clinical examinations were performed at the preschools by three dentists who had undergone the calibration exercise. To facilitate the diagnosis, each child received a kit containing a toothbrush, toothpaste and dental floss to remove bacterial plaque from the teeth under the examiner’s supervision prior to the exam. Oral examinations were performed in the knee-to-knee position with the aid of a portable lamp attached to the examiner’s head (Petzl Zoom head lamp, Petzl America, Clearfield, UT, USA). The dentists used individual cross-infection protection equipment as well as packaged, sterilized mouth mirrors (PRISMA®, Sao Paulo, SP, Brazil), Williams’ periodontal probes (WHO-621, Trinity®, Campo Mourão, PA, Brazil) and dental gauze to dry the teeth.

Dental caries was diagnosed using the ICDAS II [14], which is a scoring system ranging from 0 (absence of dental caries) to 6. Due to the epidemiological nature of the present study, code 1, that corresponds to first visual change in enamel, was not used, as drying of the teeth was performed with gauze rather than compressed air. Because it would not be possible to visually observe code 1 without compressed air, the code 1 was not used. Codes ≥ 2 were used, being: 2) distinct visual change in enamel when wet, used for white spots; 3) localised enamel breakdown (without clinical visual signs of dentine involvement); 4) underlying dark shadow from dentine; 5) distinct cavity with visible dentine; and 6)large, visible cavity in the dentin, at the base and walls affecting more than half of the surface. Codes ≥ 3 determined different degrees of cavitation. The variable dental caries was evaluated for the presence and absence in all teeth. Dental caries was when any teeth with code ≥ 2 was present. Caries on the upper incisors was recorded when at least one upper incisor received a code ≥ 2, regardless of the lesions on the posterior teeth.

Severity was evaluated using the index proposed by Hallet, O´Rourke [15], with a modification. As the original index does not include non-cavitated lesions or teeth with with spots, a code 0 was included for these situations. The classification for severity was as follow:

0 = caries free/non-cavitated lesion (white spot);

1 = low severity (1 to 5 cavitated lesions);

2 = high severity (6 or more cavitated lesions).

Malocclusion was recorded in the presence of at least one of the following conditions: deep overbite, anterior open bite, increased overjet and posterior crossbite. To measure overjet, the examiner placed the periodontal probe on the incisal surface of the maxillary central incisors parallel to the occlusal plane to determine the horizontal relation of the incisors with the teeth in centric occlusion. Overjet was dichotomized as i) 2 mm or less (normal) and ii) greater than 2 mm (increased) [16, 17]. Open bite was recorded when the anterior teeth were not in contact with the posterior teeth during occlusion [18].

The classification proposed by Andreasen [19] was used for the clinical diagnosis of traumatic dental injury (TDI): enamel fracture, enamel + dentin fracture, complicated crown fracture, extrusive luxation, lateral luxation, intrusive luxation and avulsion. A visual assessment of tooth discoloration was also performed. TDI was recorded when the child exhibited at least one of these injuries.

**Statistical analysis**

Descriptive statistics were first performed to characterize the sample. The bivariate logistic regression analysis for complex samples was used to test associations between the independent variables and the dependent variable (perceived impact on OHRQoL of preschoolers children and their families). TDI and malocclusion were controlled as variables of confusion (p<0.05).

The independents variables that had a p-value <0.20 in the bivariate analysis were selected to be included into the multivariate model. The backward stepwise procedure was used to incorporate these variables. This backward model initially incorporates all variables with p <0.20 and after testing, those who do not obtain p-value <0.05 are eliminated from the model because they are not considered statistically significant. The statistical analysis was done using ‘type of school’ to weight the analysis. For this reason, ‘type of school’ was not included in bivariate and multivariate models. Variables with a p-value < 0.05 in the adjusted analysis were maintained in the final regression model. Interactions among dental caries, TDI and malocclusion were tested using Wald’s test. Variance inflation factors were calculated to determine the existence of collinearity among the predictors in the adjusted model. The data were organized and analyzed with the aid of the Statistical Package for Social Sciences (SPSS for Windows, version 20.0, SPSS Inc, Chicago, IL, USA).

**Results**

Among the 864 preschool children selected, 843 participated in the present study, corresponding 97.56% of the total determined by the sample size calculation. The loss of 21 children was due to a lack of cooperation during the exam (n = 6), incomplete questionnaires (n = 11) and absence from preschool/daycare on the days scheduled for the clinical examinations (n = 4). Table 1 displays the socio-demographic and clinical data of the sample. The prevalence of dental caries was 66.3%.

**Table 1- Sample characterization and clinical data**

| **Variable** | **Frequency** | |
| --- | --- | --- |
|  | **N** | **%** |
| **Sex** | | |
| Female | 407 | 48.3 |
| Male | 436 | 51.7 |
| **Age** | | |
| 3 years | 275 | 32.6 |
| 4 years | 334 | 39.6 |
| 5 years | 234 | 27.8 |
| **Type of preschool** | | |
| Public | 456 | 54.1 |
| Private | 387 | 45.9 |
| **Order of child birth *** | | |
| Only child | 263 | 31.1 |
| Youngest child | 349 | 41.3 |
| Middle child | 104 | 12.3 |
| Oldest child | 123 | 14.5 |
| Absence of answers | 4 | 0.8 |
| **Household income*** | | |
| ≤ 1 Brazilian minimum salary | 442 | 54.4 |
| > Brazilian minimum salary | 362 | 42.9 |
| Absence of answers | 39 | 4.6 |
| **Caregiver’s schooling *** | | |
| ≤ 8 years of study | 388 | 46.0 |
| > 8 years of study | 452 | 53.6 |
| Absence of answers | 3 | 0.4 |
| **Caregiver’s age *** |  |  |
| ≤ 30 years | 422 | 50.0 |
| > 30 years | 403 | 47.8 |
| Absence of answers | 18 | 2.2 |
| **Number of residents in home *** | | |
| < 6 | 699 | 82.9 |
| ≥ 6 | 129 | 15.3 |
| Absence of answers | 15 | 1.8 |
| **Dental caries** |  |  |
| Present | 559 | 66.3 |
| Absent | 284 | 33.7 |
| **Severity of caries** |  |  |
| Caries free/non-cavitated lesion | 217 | 25.7 |
| Low severity | 188 | 22.3 |
| High severity | 438 | 52.0 |
| TOTAL | 843 | 100 |

Perceived impact on OHRQoL was greater among the children (32.5%) than the families (26.3%). The items with the greatest frequencies on the Child Impact section of the B-ECOHIS were “pain in the teeth” (23.1%), “had difficulty drinking hot or cold beverages” (13.0%) and “had difficulty eating some foods” (13.3%). The items with the greatest frequencies on the Family Impact section were “felt guilty” (18.5%) and “been upset” (14.9%) (Table 2).

Table 2- Frequency of perceived impact on child, family and B-ECOHIS items

|  | **Frequency of Impact** | |  |  |
| --- | --- | --- | --- | --- |
| **Domains, Items** | **Yes** | **No** | **Don’t know** | **Total** |
|  | **N(%)** | **N(%)** | **N(%)** | **N (%)** |
| **Impact on child** | 274(32.5) | 569(67.5) |  |  |
| Report of pain in teeth | 195(23.1.) | 629(74.6) | 19(2.3) | 843(100) |
| Had difficulty drinking hot or cold beverages | 110(13.0) | 725(86.0) | 8(0.9) | 843(100) |
| Had difficulty eating some foods | 112(13.3) | 722(85.6) | 9(1.1) | 843(100) |
| Had difficulty pronouncing words | 66(7.8) | 752(89.2) | 25(3.0) | 843(100) |
| Missed preschool | 34(4.0) | 802(95.1) | 7(0.8) | 843(100) |
| Had difficulty sleeping | 56(6.6) | 781(92.6) | 6(0.7) | 843(100) |
| Been irritable or frustrated | 95(11.3) | 742(88.0) | 6(0.7) | 843(100) |
| Avoided smiling or laughing | 26(3.1) | 809(96.0) | 8(0.9) | 843(100) |
| Avoided speaking | 27(3.2) | 809(96.0) | 7(0.8) | 843(100) |
| **Impact on family** | 222(26.3) | 621(73.7) |  |  |
| Been upset | 126(14.9) | 708(84.0) | 9(1.1) | 843(100) |
| Felt guilty | 156(18.5) | 678(80.4) | 9(1.1) | 843(100) |
| Missed work | 56(6.6) | 781(92.6) | 6(0.7) | 843(100) |
| Financial problem | 46(5.5) | 785(93.1) | 12(1.4) | 843(100) |

In Table 3, we have the result of bivariate logistic regression models for the impact of dental caries in the quality of life of preschool children, their families and the independent variables.

**Table 3- Bivariate logistic regression models for the perceived impact of dental caries in the quality of life of pre-school children, their families and the independent variables.**

| **Variable** | **Perceived Impact on child** | | **Bivariate Unadjusted OR** | | **Perceived Impact on family** | | **Bivariate Unadjusted OR** | |
| --- | --- | --- | --- | --- | --- | --- | --- | --- |
|  | **Yes** | **No** | **p** | **(95% CI)** | **Yes** | **No** | **p** | **(95%CI)** |
|  | n(%) | n (%) |  |  | n (%) | n (%) |  |  |
| **Child’s sex** |  |  |  |  |  |  |  |  |
| Female | 274(67.3) | 133(32.7) | 0.475 | 1.136(0.800-1.612) | 300(73.7) | 107(26.3) | 0.657 | 1.087(0.752-1.572) |
| Male | 295(67.7) | 141(32.3) | - | 1 | 321(73.6) | 115(26.4) |  | 1 |
| **Child’s age** |  |  |  |  |  |  |  |  |
| 3 years | 204(74.2) | 71(25.8) | - | 1 | 209(76.0) | 66(24.0) | - | 1 |
| 4 years | 239(71.6) | 95(28.4) | 0.787 | 1.060(0.696-1.612) | 249(74.6) | 85(25.4) | 0.939 | 1.349(0.839-2.169) |
| 5 years | 126(53.8) | 108(46.2) | 0.003 | 2.045(1.284-3.258) | 163(69.7) | 71(30.3) | 0.216 | 0.984(0.645-1.500) |
| **Caregiver’s schooling** | | | | | | | | |
| ≤ 8 years | 234(60.3) | 154(39.7) | <0.001 | 1.871(1.320-0.654) | 270(69.6) | 118(30.4) | 0.005 | 1.692(1.171-2.445) |
| > 8 years | 332(73.5) | 120(26.5) | - | 1 | 348(77.0) | 104(23.0) | - | 1 |
| **Number of residents in home** | | | | | | | | |
| < 6 | 482(69.0) | 217(31.0) | - | 1 | 522(74.7) | 177(25.3) | - | 1 |
| ≥ 6 | 76(58.9) | 53(41.1) | 0.042 | 1.569(1.017-2.420) | 86(66.7) | 43(33.3) | 0.074 | 0.513(0.961-2.381) |
| **Caregiver’s age** | | | | | | | | |
| ≤ 30 years | 283(67.1) | 139(32.9) | 0.357 | 1.183(0.827-1.690) | 302(71.6) | 120(28.4) | 0.006 | 1.683(1.158-2.447) |
| > 30 years | 277(68.7) | 126(31.3) | - | 1 | 309(76.6) | 94(23.3) | - | 1 |
| **Household income** | | | | | | | | |
| ≤ 1 min. Salary | 265(60.0) | 177(40.0) | <0.001 | 1.977(1.953-2.890) | 310(70.1) | 132(29.9) | 0.098 | 1.392(0.946-2.048) |
| > 1 min. Salary | 273(75.4) | 89(24.6) | - | 1 | 279(77.1) | 83(22.9) | - | 1 |
| **Order of child birth** | | | | | | | | |
| Only child | 203(77.2) | 60(22.8) | - | 1 | 208(79.1) | 55(20.9) | - | 1 |
| Oldest child | 73(59.3) | 50(40.7) | <0.001 | 2.797(1.584-4.098) | 84(68.3) | 39(31.7) | 0.005 | 2.340(1.287-4.257) |
| Youngest child | 226(64.8) | 123(35.2) | 0.043 | 1.577(1.013-2.453) | 251(71.9) | 98(28.1) | 0.137 | 1.399(0.898-2.180) |
| Middle child | 65(62.5) | 39(37.5) | 0.091 | 1.632(0.924-2.883) | 76(73.1) | 28(26.9) | 0.306 | 1.360(0.754-2.452) |
| **Perception of general health** | | | | | | | | |
| Good | 477(70.1) | 203(29.9) | - | 1 | 523(76.9) | 157(23.1) | - | 1 |
| Poor | 88(55.3) | 71(44.7) | <0.001 | 2.050(1.385-3.034) | 97(61.0) | 62(39.0) | <0.001 | 2.324(1.549-3.486) |
| **Perception of oral health** | | | | | | | | |
| Good | 439(78.4) | 121(21.6) | - | 1 | 473(84.5) | 87(15.5) | - | 1 |
| Poor | 129(45.7) | 153(54.3) | <0.001 | 4.372(2.987-6.401) | 147(52.1) | 135(47.9) | <0.001 | 4.876(3.254-7.304) |
| **Dental Pain** | | | | | | | | |
| Yes | 111(87.4) | 16(12.6) | <0.001 | 67.525(27.995-162.871) | 26(32.9) | 53(67.1) | <0.001 | 8.023(3.756-17.082) |
| No | 11(13.9) | 68(86.1) | - | 1 | 103(81.1) | 24(18.9) | - | 1 |
| **Dental caries** | | | | | | | | |
| Present | 332(59.4) | 227(40.6) | <0.001 | 3.789(2.460-5.834) | 371(66.4) | 188(33.6) | <0.001 | 3.399(1.972-5.858) |
| Absent | 237(83.5) | 47(16.5) | - | 1 | 250(88.0) | 34(12.0) | - | 1 |
| **Caries on maxillary incisor** | | | | | | | | |
| Yes | 173(55.1) | 141(44.9) | <0.001 | 2.530(1.769-3.617) | 197(62.7) | 117(37.3) | <0.001 | 2.418(1.659-3.527) |
| No | 396(74.9) | 133(25.1) | - | 1 | 424(80.2) | 105(19.8) | - | 1 |
| **Severity of caries** | | | | | | | | |
| Caries free/non-cavitated | 361(82.4) | 77(17.6) | - | 1 | 384(87.7) | 54(12.3) | - | 1 |
| Low severity | 132(70.2) | 56(29.8) | 0.006 | 2.048(1.233-3.400) | 138(73.4) | 50(26.6) | <0.001 | 2.792(1.617-4.821) |
| High severity | 76(35.0) | 141(65.0) | <0.001 | 8.190(5.203-12.890 | 99(45.6) | 118(54.4) | <0.001 | 8.408(5.160-13.702) |
| **TDI** | | | | | | | | |
| Yes | 183(66.3) | 93(33.7) | 0.619 | 1.102(0.751-1.617) | 112(40.6) | 164(59.4) | 0.052 | 1.132(0.751-1.708) |
| No | 371(69.5) | 163(30.5) | - | 1 | 216(40.4) | 318(59.6) | - | 1 |
| **Malocclusion** | | | | | | | | |
| Present | 353(66.5) | 178(33.5) | 0.923 | 1.018(0.706-1.468) | 222(41.8) | 309(58.2) | - | 1 |
| Absent | 213(69.2) | 95(30.8) | - | 1 | 128(41.6) | 180(58.4) | 0.900 | 1.025(0.697-1.509) |

|  |  | |  | | |  | |  | | | | |
| --- | --- | --- | --- | --- | --- | --- | --- | --- | --- | --- | --- | --- |
|  |  | |  | |  |  | |  | |  |  |  |
|  |  | |  | |  |  | |  | |  |  |  |
|  | | | | | | |  | | | | | |
|  |  | |  | |  |  | |  | |  |  |  |
|  |  | |  | |  |  | |  | |  |  |  |
|  |  |  | | |  | |  | | | | | |
|  |  | |  | |  |  | |  | |  |  |  |
|  |  | |  | |  |  | |  | |  |  |  |
|  |  | |  | |  |  | |  | |  |  |  |
|  | |  | | |  | |  | | | | | |
|  |  | |  | |  |  | |  | |  |  |  |
|  |  | |  | |  |  | |  | |  |  |  |
|  | | | | |  | |  | | | | | |
|  |  | |  | |  |  | |  | |  |  |  |
|  |  | |  | |  |  | |  | |  |  |  |
|  | |  | | |  | |  | | | | | |
|  |  | |  | |  |  | |  | |  |  |  |
|  |  | |  | |  |  | |  | |  |  |  |
|  | |  | | |  | |  | | | | | |
|  |  | |  | |  |  | |  | |  |  |  |
|  |  | |  | |  |  | |  | |  |  |  |
|  | |  | | |  | |  | | | | | |
|  |  | |  | |  |  | |  | |  |  |  |
|  |  | |  | |  |  | |  | |  |  |  |
|  |  | |  | |  |  | |  | |  |  |  |
|  |  | |  | |  |  | |  | |  |  |  |
|  | | | | |  | |  | | | | | |
|  |  | |  | |  |  | |  | |  |  |  |
|  |  | |  | |  |  | |  | |  |  |  |
|  | | | | |  | |  | | | | | |
|  |  | |  | |  |  | |  | |  |  |  |
|  |  | |  | |  |  | |  | |  |  |  |
|  |  | |  | |  |  | |  | |  |  |  |
|  |  | |  | |  |  | |  | |  |  |  |
|  |  | |  | |  |  | |  | |  |  |  |
|  | | |  | |  |  | |  | |  |  |  |
|  |  | |  | |  |  | |  | |  |  |  |
|  |  | |  | |  |  | |  | |  |  |  |
|  | | | | |  | |  | | | | | |
|  |  | |  | |  |  | |  | |  |  |  |
|  |  | |  | |  |  | |  | |  |  |  |
|  | |  | | |  | |  | | | | | |
|  |  |  |  |  | |  |  |  |  | | | |
|  |  |  |  |  | |  |  |  |  | | | |
|  |  |  |  |  | |  |  |  |  | | | |
|  | |  |  |  | |  |  |  |  | | | |
|  |  |  |  |  | |  |  |  |  | | | |
|  |  |  |  |  | |  |  |  |  | | | |
|  | |  |  |  | |  |  |  |  | | | |
|  |  |  |  |  | |  |  |  |  | | | |
|  |  |  |  |  | |  |  | |  |  | | --- | --- | | |  |  | | --- | --- | | | | |

In the final model of the logistic regression analysis for complex samples, the following variables were associated with the prevalence of perceived impact on OHRQoL of children, order of birth of the child, being the middle child (OR: 10.107, 95%CI: 2.008-50.869) and youngest child (OR: 3.276, 95%CI: 1.048-10.284) and dental pain (OR: 84.477, 95%CI: 33.076-215.759) (Table 4). The following variables was associated with the prevalence of perceived impact on OHRQoL family: Poor perception of oral health was significant predictor of the perceived impact on OHRQOL for family (OR=7.397, 95%CI: 2.190-24.987) (Table 4).

**Table 4- Multivariate logistic regression models for the impact of dental caries in the quality of life of pre-school children, their families and the independent variables.**

| **Variable** | **Impact on Child** | | **Multivariate adjusted OR** | |
| --- | --- | --- | --- | --- |
|  | **Yes** | **No** | **p** | **(95% IC)** |
|  | **n(%)** | **n(%)** |  |  |
| **Order of child birth** |  |  |  |  |
| Only child | 203(77.2) | 60(22.8) | - | 1 |
| Oldest child | 73(59.3) | 50(40.7) | 0.050 | 4.068(1.003-16.500) |
| Youngest child | 226(64.8) | 123(35.2) | 0.042 | 3.276(1.048-10.284) |
| Middle child | 65(62.5) | 39(37.5) | 0.005 | 10.107(2.008-50.869) |
| **Dental Caries** |  |  |  |  |
| Present | 332(59.4) | 227(40.6) | 0.275 | 2.610(0.403-14.700) |
| Absent | 237(83.5) | 47(16.5) | - | 1 |
| **Severity of caries** |  |  |  |  |
| Free of caries and white spot | 361(82.4) | 77(17.6) | - | 1 |
| Low | 132(70.2) | 56(29.8) | 0.483 | 0.596(0.139-2.552) |
| High | 76(35.0) | 141(65.0) | 0.531 | 0.590(0.112-3.103) |
| **Variable** | **Impact on Family** | | **Multivariate adjusted OR** | |
|  | **Yes** | **No** | **p** | **(95%IC)** |
|  | **n (%)** | **n(%)** |  |  |
| **Perception of oral health** |  |  |  |  |
| Good | 439(78.4) | 121(21.6) | - | 1 |
| Poor | 129(45.7) | 153(54.3) | <0.001 | 7.397(2.190-24.987) |
| **Dental Caries** |  |  |  |  |
| Present | 371(66.4) | 188(33.6) | 0.292 | 2.649(0.429-16.340) |
| Absent | 250(88.0) | 34(12.0) | - | 1 |
| **Severity of caries** |  |  |  |  |
| Free of caries and white spot | 384(87.7) | 54(12.3) | - | 1 |
| Low | 138(73.4) | 50(26.6) | 0.634 | 1.558(0.248-9.776) |
| High | 99(45.6) | 118(54.4) | 0.230 | 3.453(0.454-26.270) |

|  | |  | |  | |
| --- | --- | --- | --- | --- | --- |
|  | |  |  |  |  |
|  | |  |  |  |  |
|  | | | | | |
|  | |  |  |  |  |
|  | |  |  |  |  |
|  | |  |  |  |  |
|  | |  |  |  |  |
|  | | | | | |
|  | |  |  |  |  |
|  | |  |  |  |  |
|  | | | | | |
|  | |  |  |  |  |
|  | |  |  |  |  |
|  | | | | | |
|  | |  |  |  |  |
|  | |  |  |  |  |
|  | |  |  |  |  |
|  | |  | |  | |
|  |  | |  |  |  |
|  |  | |  |  |  |
|  | | | | | |
|  | |  |  |  |  |
|  | |  |  |  |  |
|  | | | | | |
|  | |  |  |  |  |
|  | |  |  |  |  |
|  | | | | | |
|  | |  |  |  |  |
|  | |  |  |  |  |
|  | |  |  |  |  |

**Discussion**

The prevalence of dental caries was high in the present sample (66.3%). The literature describes prevalence rates ranging from 46 to 53% in developing countries [3, 20-22] and 22 to 32% in industrialized countries [23, 24]. These differences may be influenced by the characterization of the sample, the different methods employed, the region in which the study was carried out and the index used for the diagnosis of caries. The studies cited used the DMFT index [20-24], whereas the ICDAS-II and the index the gravity [15] were employed in the present investigation, which includes the initial stages of tooth decay (white spots) and likely contributed to the higher prevalence rate. However, although ICDAS-II may contribute to a higher prevalence of caries compared to DMFT index, the present study did not used the code 1, which can account for an underestimation of caries in the present sample. By contrast, as code 1 corresponds to white spots in enamel when dried by compressed air, visually detectable the dentist in the dental office, but not by lay persons. For this reason, the modification on the index may have little impact over the results, as the dependent variable is perceived impact on OHRQoL, and parents would hardly perceive a code 1 by visual inspection. The ICDAS-II is considered to have greater sensitivity and specificity due to the fact that it involves the early stages of dental caries through to extensive cavities that reach the dentin [14, 25]. Another study used the same criterion of diagnosis for dental caries and found results similar to our study [26]. Also, the original index of severity of caries includes low and high severity. The inclusion of a modification (code 0) was included to be used as reference category, as it is expected that caries free children or children with white spots would account to absence of perceived impact on OHRQoL, whereas the presence of cavitated lesions are more easily perceived by parents.

The perceived impact on OHRQoL was greater among the children than in the families. The comparison between the perceived impact of the child and the family has also been reported in previous studies [3,4,27]. While one study found similar results (perceived impact of the child 33.5% and the perceived impact of the family 22.9%) [3], other study shows a marked difference (perceived impact of the child 69.3% and perceived impact of the family30,7%) [27]. This divergence can be explained by differences in sample analyzed and the methods employed. The study with larger differences between the results, children were recruited from health services [27]. The present study was representative from private and public preschools. This may have influenced the results. A sample from health services is a high selective population that may have perceived impact on OHRQoL, since they have already searched for dental services. In representative samples, the sample was distributed at random, and it is expected to find families who have searched for dental treatment and families who have not. The higher perceived impact among the children in comparison to the families may be explained by an initial lack of perception on the part of the parents, leading to dental pain and discomfort stemming from the absence of treatment [28].Furthermore, these results lead us to think that perhaps these children suffering with oral health problems have a greater perception of the implications and consequences of this impact on OHRQoL.

Analyzing the prevalence of the B-ECOHIS items, the most frequent impacts were "reported pain", “had difficulty drinking hot or cold beverages” and "had difficulty eating some foods" in the Child Impact Section and "felt guilty" and “been upset” in the Family Impact section. These findings are similar to data reported in previous studies [28-30]. These items may be the most cited because they affect sleep, nutrition and school attendance and require time from parents/caregivers and family members, thereby contributing a greater perceived impact on both the child and family [6].

Being the middle child and being the youngest child led to an approximately tenfold and threefold greater chance, respectively, of having perceived impact on OHRQoL among the preschool children. This may be explained by the fact that financial resources and attention from parents/caregivers are shared among siblings as more children are born in the family [31, 32]. However, the p-value for the category youngest child was very close to the limit of significance (0.042).

Parents that reported that their children had history of dental pain had about 84-fold chance of reporting perceived impact on OHRQoL among the children. This factor is a major reason for seeking dental treatment at this stage of life [33], as parents/caregivers recognize oral problems in their children when the pain occurs [34], so maybe, that’s why the severity of caries and dental caries have not been recognized as a predictor of perceived impact on OHRQoL. Other studies of different age group [4] and using different evaluation tools [5, 35] also reported that dental pain is the most frequent specific cause of perceived impact on OHRQoL [36]. These results demonstrated that dental pain can be the most important factor, even in the presence of high severity of dental caries, about the perceived impact on quality of life, regardless of age and questionnaires used. It seems that for parents, dental pain means need for dental care, whereas a child can undergo without dental treatment even in the presence of dental caries, unless it turns into pain.

Parent’s/caregiver’s that perceived their child’s oral health as poor had about seven-fold greater chance of reporting impact on OHRQoL on the family compared to those parent’s/caregiver’s that reported perceptions of their child’s oral health as good. Indeed, this variable is an important indicator of a perceived impact on quality of life, as the maintenance of a child’s oral health depends on the knowledge of parents/caregivers regarding this issue [33, 37-38]. Studies have shown that perceptions of parents are associated with clinical characteristics, such as children with tooth decay and dental pain reports are more likely to have your oral health status classified as poor [33-39,40]. Parents / caregivers are responsible for preventing oral health problems [1]. In addition, the perception of poor oral health is associated with need for dental treatment in preschool children [40].

The present study has the inherent limitations of the cross-sectional design, such as the lack of temporality. However, the inferences of the cross-sectional study can establish the direction of the associations, as presented by the present study. Data collected through questionnaires can have biased results. The use of a validated questionnaires can be a useful strategy to minimize bias. The execution of a pilot studywas also implemented to test the instruments before the main study could be conducted. Longitudinal studies are needed to evaluate how individuals perceived OHRQoL over time. The broad confidence interval regarding "Dental Pain" (Table 3 and 4) can be considered a limitation of the present study. In such cases, it is more difficult to determine a precise effect size and there may be some uncertainty in the results. However, there may be enough precision to make decisions about the usefulness of an intervention. This factor may account to some heterogeneity of the sample [41].

**Conclusions**

The order of birth of the child, being the middle child and youngest son, and a history of dental pain were found to be indicators of perceived impact on OHRQoL among preschool children and parent’s/caregiver’s perception of their child’s oral health as poor was found to be indicators of impact on OHRQoL among their families.Dental caries was not associated to perceived impact on OHRQoL of children or families. The evaluation of OHRQoL can help health administrators in the planning and decision-making process regarding the implementation of prevention and control measures at oral health services. It is important to be aware of the risk factors that perceived impact the quality of life preschoolers in order to facilitates better oral health guidance for parents / caregivers and to promote and to incentive the search for preventive dental care for this group.

**References**

1. Arora A, Scott JA, Bhole S, Do L, Schwarz E,Blinkhorn AS. Early childhood feeding practices and dental caries in preschool children: a multi-centre birth cohort study. BMC Public Health. 2011;11:28.
2. Alkarimi HA, Watt RG, Pikhart H, Jawadi AH, Sheiham A, Tsakos G. Impact of treating dental caries on schoolchildren’s anthropometric, dental, satisfaction and appetite outcomes: a randomized controlled trial. BMC Public Health. 2012;12:706.
3. Martins-Júnior PA, Vieira-Andrade RG, Corrêa-Faria P, Oliveira-Ferreira F, Marques LS, Ramos-Jorge ML.Impact of early childhood caries on the oral health-related quality of life of preschool children and their parents. Caries Res. 2013; 47:211-218.
4. Leal SC, Bronkhorst EM, Fan M, Frencken JE.Untreated cavitated dentine lesions: impact on children’s quality of life. Caries Res. 2012 46:102-106.
5. Bianco A, Fortunato L, Nobile CG, Pavia M. Prevalence and determinants of oral impacts on daily performance: results from a survey among school children in Italy. Eur J Public Health. 2010; 20:595-600.
6. Boeira GF, Correa MB, Peres KG, Peres MS, Santos IS, Matijasevich A, et al.Caries is the main cause for dental pain in childhood: findings from a birth cohort. [Caries Res](http://www.ncbi.nlm.nih.gov/pubmed/22813889). 2012; 46:488-495.
7. Pahel BT, Rozier RG, Salde GD. Parental perceptions of children’s oral health: The Early Childhood Oral Health Impact Scale (ECOHIS). Health Qual Life Outcomes. 2007;5:6.
8. Barbosa TS, Gavião MB. Oral health-related quality of life in children: Part II. Effects of clinical oral health status. A systematic review. Int J Dent Hyg. 2008; 6;100-107.
9. Tesch FC, Oliveira BH, Leão A.Semantic equivalence of the Brazilian version of the Early Chilhood Oral Health Impact Scale. Cad. Saude Publica. 2008; 4:1897-1909.
10. Instituto Brasileiro de Geografia e Estatística (IBGE): Primeiros resultados do Censo2010.(2010) Available: http://www.censo2010.ibge.gov.br/dadosdivulgados/index.php?uf=25. Accessed on 3 August 2012.
11. David J, Astrom A, Wang NJ. Factors associated with traumatic dental injuries among 12-year-old schoolchildren in South India. Dent Traumatol. 2009; 25:500-505.
12. Altman DG. Practical statistics for medical research. Chapman and Hall: London, England, 2006.
13. Scarpelli AC, Paiva SM, Viegas CM, Carvalho AC, Ferreira FM, Pordeus IA.Oral health-related quality of life among Brazilian preschool children. Community Dent Oral Epidemiol. 2013; 41:336-344.
14. Ismail AI, Sohn W, Tellez M, Amaya A, Sen A, Hasson H, et al. The International Caries Detection and Assessment System (ICDAS): an Integrated System for Measuring Dental Caries. Community Dent Oral Epidemiol. 2007; 35:170-178.
15. Hallett KB, O’rourke PK. Pattern and severity of early childhood caries.Community Dent Oral Epidemiol. 2006;34:25–35.
16. Foster TD, Hamilton MC. Occlusion in the primary dentition. Study of children at 21 to 3 years of age. Br Dent J. 1969; 126:76-79.
17. Grabowski R, Stahl F, Gaebel M, Kundt, G. Relationship between occlusal findings and orofacial myofunctional status in primary and mixed dentition. Part I: Prevalence of malocclusions. J Orofac Orthop. 2007; 68:26-37.
18. World Health Organization (WHO): Oral Health Surveys. Basic Methods, 1997.
19. Andreasen JO, Andreasen FM, Andersson L. Textbook and Color Atlas of Traumatic Injuries to the Teeth, Munskgaard International Publishers: Copenhagen, Denmark, 2007.
20. Shang XH, Li DL, Huang Y, Chen H, Sun RP. Prevalence of dental caries among preschool children in Shanghe County of Shandong Province and relevant prevention and treatment strategies. Chin Med J. 2008;20:2246-2249.
21. Cleaton-Jones P, Williams S, Green C, Fatti P. Dental caries rates in primary teeth in 2002, and caries surveillance trends 1981-2002, in a South African city. Community Dent Health. 2008,2:79-83.
22. Simratvir M, Moghe GA, Thomas AM, Singh N, Chopra S. Evaluation of caries experience in 3-6-year-old children, and dental attitudes amongst the caregivers in the Ludhiana city. J Indian Soc Pedod Prev Dent. 2009; 27:164-169.
23. Pitts NB, Boyles J, Nugent ZJ, Thomas N, Pine CM. The dental caries experience of 5-year-old children in Great Britain (2005/6). Surveys co-ordinated by the British Association for the study of community dentistry. Community Dent Health. 2007; 1:59-63.
24. Campus G, Solinas G, Strohmenger L, Cagetti MG, Senna A, Minelli L, et al. National pathfinder survey on children’s oral health in Italy: pattern and severity of caries disease in 4-year-olds. Caries Res. 20092:155-162.
25. Cadavid AS, Lince CMA, Jaramilo MC. Dental caries in the primary dentition of a Colombian population according to the ICDAS criteria. Braz Oral Res. 2010;24:211-216.
26. Amorim RG, Figueiredo MJ, Leal SC, Mulder J, Frencken JE. Caries experience in a child population in a deprived area of Brazil, using ICDAS II. Clin Oral Investig. 2012; 16:513-522.
27. Aldrigui JM, Abanto J, Carvalho TS, Mendes FM, Wanderley MT, Bönecker M, et al. Impact of traumaticdentalinjuries and malocclusions on quality of life of youngchildren. Health Qual Life Outcomes. 201;1 9:78.
28. Lee GHM, McGrath C, Yiu CKY, King NM. A comparison of a generic and oral health–specific measure in assessing the impact of early childhood caries on quality of life. Community Dent Oral Epidemiol. 2010 38:333-9.
29. Li S, Veronneau J, Allison PJ. Validation of a Frenchlanguage version of the Early Childhood Oral HealthImpact Scale (ECOHIS). Health Qual Life Outcomes. 2008; 6:9.
30. Lee GHM, McGrath C, Yiu CKY, King NM. Translation and validation of a Chinese language version of the Early Childhood Oral Health Impact Scale (ECOHIS). Int J Paediatr Dent. 2009;19:399-405.
31. Hertwig R, Davis JN, Sulloway FJ. Parental investment: how an equity motive can produce inequality.Psychol Bull. 2002;128:728-745. [

](http://www.hqlo.com/sfx_links?ui=1477-7525-12-55&bibl=B36)
32. Hearton TB, Forste R, Hoffmann JP, Flake D. Cross-national variation in family influences on child health.Soc Sci Med. 2005; 60:97-108.
33. Wandera M, Kayondo J, Engebretsen IM, Okullo I, Astrom AN. Factorsassociated with caregivers’ perception of children’s health and oralhealth status: a study of 6- to 36-month-olds in Uganda. Int J PaediatrDent. 2009;19:251-262.
34. Kosmala-Anderson J, Wallace LM. Breastfeeding works: The role of employers in supporting women who wish to breastfeed and work in four organizations in England. J Public Health Med. 2006; 28:183–191.
35. Easton JA, Landgraf JM, Casamassimo PS, Wilson S, Ganzberg S. Evaluation of a generic quality of life instrument for early childhood caries-related pain. Community Dent Oral Epidemiol. 2008; 36:5:434-40.
36. Moura-Leite FR, Ramos-Jorge J, Ramos-Jorge ML, Paiva SM, Vale MP, Pordeus IA. Impact of dental pain on daily living of Five-year-old preschool children: prevalence and associated factors. Eur Arch Paediatr Dent.2011; 6:293-297.
37. Camargo MB, Barros AJ, Frazão P, Matijasevich A, Santos IS,Peres MA.Predictors of dental visits for routine check-ups and for the resolution of problems among preschool children. Rev. Saude Publica. 2012;46:87-97.
38. Thikkurissy S, Allen PH, Smiley MK, Casamassimo PS. Waiting for the pain to get worse: characteristics of a pediatric population with acute dental pain. Pediatr Dent. 2012;34:289-294.
39. Piovesan C, Marquezan M, Kramer PF, Bönecker M, Ardenghi TM Socioeconomic and clinical factors associated with caregivers’ perceptions of children’s oral health in Brazil. Community Dent Oral Epidemiol. 2011; 39:260-267.
40. Talekar BS, Rozier RG, Slade GD, Ennett ST. Parental perceptions of their preschool-aged children’s oral health. J Am Dent Assoc. 2005; 136:364-372.
41. Higgins JPT, Green S. Cochrane Handbook for Systematic Reviews of Interventions Version 5.1.0 [updated March 2011]. The Cochrane Collaboration. 2011; Available from www.cochrane-handbook.org. Accessed: 18 November 2014.
